# Molecular Identification of Diaspididae and Elucidation of Non-Native Species Using the Genes 28s and 16s

**DOI:** 10.3390/insects5030528

**Published:** 2014-07-03

**Authors:** Alexander M. Campbell, Andrew J. Lawrence, Caleb B. Hudspath, Matthew E. Gruwell

**Affiliations:** Penn State Erie, The Behrend College, 4701 College Dr, Erie, PA 16563, USA; E-Mails: ajl5131@psu.edu (A.J.L.); caleb.hudspath@rvu.edu (H.C.)

**Keywords:** *Diaspididae*, armored scale insects, phylogenetics, molecular barcoding, invasive species, cryptic species

## Abstract

Armored scale insects pose a serious threat to habitat conservation across the globe because they include some of the most potent invasive species in the world. They are such a serious concern because their basic morphology, small size, and polyphagous feeding habits often allow them to exist undetected by growers and quarantine experts. In order to provide a potential solution to the problem, we have attempted to elucidate the effectiveness of molecular identification techniques using ribosomal 28s and endosymbiotic 16s rRNA. Sequence data was obtained from many field-collected insects to test the feasibility of identification techniques. A protocol for quick species determination based on sequence data is provided.

## 1. Introduction

The introduction of invasive scale insects has caused serious economic and ecological disasters documented. Some of the largest problems have included the introduction of *Icerya purchasi* to the USA resulting in the near collapse of the citrus industry and the invasion of cassava mealybug *Phenacoccus manihoti* to West Africa threatening a food staple of 200 million people [1]. It is estimated that there are 1019 scale insect species in the United States of which approximately 255 are invasive [2]. Of the invasive species, 75% are considered pests compared to only 7% of indigenous scales that are considered pests [2]. The group of scale insects that potentially present the largest threat are the armored scale insects, Diaspididae. According to current estimates, there are approximately 132 invasive armored scale insects in the United States alone [2].

Diaspididae are ecological and agricultural pests that cause serious damage to native plants by excessively feeding on phloem and quickly reproducing to large population sizes [3]. They are called armored scales because of the waxy secretion the female extrudes via her pygidium, which creates a protective covering [4]. Diaspidids display extreme sexual dimorphism, however, males are difficult to collect because of their brief time as adults and the lack of males in parthenogenetic species [3,5,6]. Due to the lack of males, all identification is based on female morphology. Female armored scale insects are entirely sessile as adults and will spend their lives underneath their scale covering feeding on the host plant [7]. They lack most distinguishable morphological characteristics such as a distinctive head, thorax, and abdomen, and their eyes and antennae are primitive [8]. The lack of distinctive morphology causes extremely problematic identification of invasive or pest species for non-expert scale insect taxonomists [4]. 

Currently, Diaspididae comprises nearly 2500 described species [9], some of which are extremely destructive [10]. Many scales are invasive species, and their small size, problematic detection and difficult identification poses a serious challenge to customs and quarantine workers as well as conservation biologists worldwide [1,2,11]. Polyphagy further exacerbates the problem of becoming serious invasive species as some armored scales can feed on up to 100 host families [12]. Evidence of numerous morphologically cryptic species among diaspidids increases the issues surrounding identification, and phylogenetic data has shown inconsistencies in previous methods of armored scale classification [13,14,15,16]. This combined with the fact that armored scales are potent agricultural pests has made the need for effective scale insect identification dire.

One critical part of the solution to growing problems with invasive and pest species among armored scale insects will require accurate species level identification. Current measures to identify scales based on morphology are becoming ineffective due to the lack of taxonomic experience among new scientists and the continued retirement of scale insect taxonomy experts [1]. Despite this, DNA sequencing and phylogenetic analysis has opened up a new mode of molecular identification for armored scale insects [13,15]. This study is designed to determined the efficacy of molecular identification of armored scale insects using 28s rRNA gene and bacterial 16s rRNA from the armored scale insect primary endosymbiont Candidatus *Uzinura diaspidicola* [17]. Provided is a protocol to allow molecular identification of unknown species.

## 2. Experimental Section

Samples of armored scales were obtained in the summer of 2010 and 2011 from the southeastern United States including Florida, Georgia, Alabama, Mississippi, Louisiana, Missouri, Oklahoma and Texas. Genomic DNA was extracted using a modified DNeasy Kit (Qiagen, Valencia, CA, USA) protocol in which a single specimen is punctured in a location that is not critical for microscopic identification to release DNA and preserve the exoskeleton for slide mounting [13,14]. Mounted scale insects were examined to confirm molecular identification and are currently stored in the Penn State Behrend Entomology Collection.

PCRs were performed on a TC-300G thermocycler (Techne, Minneapolis, MN, USA) using the typical three-step PCR protocol with a 52 °C annealing temperature for 16S rRNA bacterial loci and 48 °C for 28S rRNA insect loci. 16S amplification was done using the following two primers, the universal forward primer 27F-AGAGTTTGATCMTGGCTCAG and a reverse primer designed from Candidatus *Uzinura diaspidicola* a1271DIASP-CATTGTAGCACGTGTGTAGCCCAAG [17]. 28S amplification was completed using universal insect 28S rRNA primers, 28S3a-AGTACGTGAAACCGTTCAGG and 28Sb-TCGGAAGGAACCAGCTAC. For both loci, twenty‑five microliter reactions contained, 12 µL Hot Start Taq Mastermix (Amresco), 1 µL each primer, 1–3 µL genomic DNA and 10–12 µL ultrapure water. All PCRs were run with negative controls and results were visualized via agarose gel electrophoresis.

Cleaned PCR products were sent to the High Throughput Genomics Center (Seattle, WA, USA) where they underwent Sanger sequencing. Sequences were then trimmed and annotated using the software package Geneious (Biomatters Ltd., Aukland, New Zealand). The original 28s and 16s sequences were combined with forty and thirty-six sequences with E-values less than 10^−20^ taken from Genbank, respectively [18]. Original data has been uploaded to Genbank and accession numbers are available in Table 1. Genbank sequence accession numbers are provided in Table 2. Phylogenetic trees using Bayesian analysis were created where each tree was run for 10 million generations with a subsampling frequency of 100,000 using Geneious software and the MrBayes plugin [19]. 

## 3. Results and Discussion

**Figure 1:**
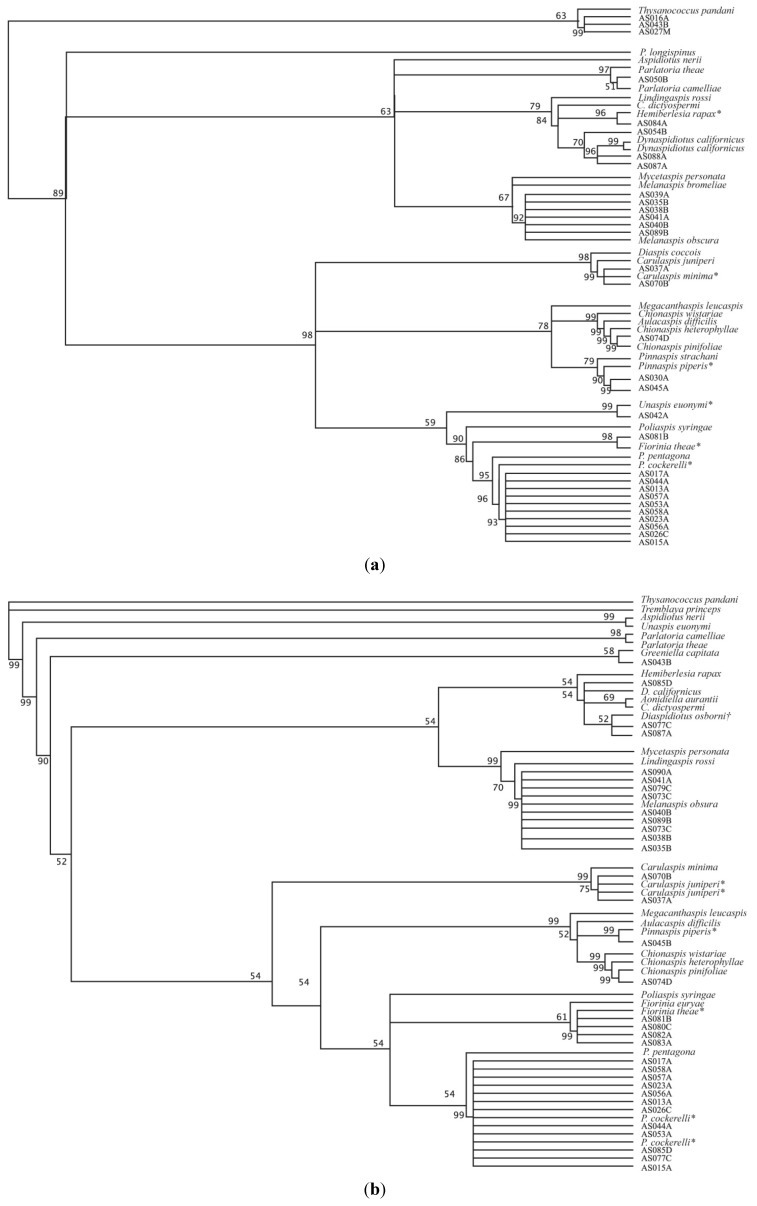
(**a**) Phylogeny of 28s utilizing Bayesian analysis. AS--- numbers indicate specimen sample numbers to correlate with collections at the Behrend Entomology Collection. Taxa provided with species names are from insects previously identified and sequenced and Genbank accession numbers are found in Table 2. An asterisk (*) indicates invasive status. Branch label indicates posterior probabilities. (**b**) Phylogeny of 16s utilizing Bayesian analysis. AS--- numbers indicate specimen sample numbers to correlate with collections at the Behrend Entomology Collection. Taxa provided with species names are from insects previously identified and sequenced and Genbank accession numbers are found in Table 2. An asterisk * indicates invasive status and a cross † indicates unknown invasive status. Branch label indicates posterior probabilities.

Results of phylogenetic tests are displayed in Figure 1a,b. Inconsistencies in species identification between the two genes were seen in samples AS037 and AS087 though the AS087 16s posterior probability is insignificant and generic ID is consistent in both cases. Species invasiveness is listed with identification in Table 1 according to Miller *et al.*, 2005 [10]. Seven different invasive species were detected in our samples of the eleven significant species detected. Samples AS013, AS015, AS017, AS023, AS026, AS044, AS053, AS056, AS057, and AS058 were identified as *Pseudalacaspis cockerelli*. Species AS030 and AS045 were identified as *Pinaspis piperis*. Samples AS035, AS038, AS039, AS040, AS041, AS073, AS079, AS089, and AS090 were identified as *Melanaspis obscura*. Specimen AS042 was identified though a 28s sequence as *Unaspis euonymi*. AS050 was identified as *Parlatoria* sp. via 28s phylogeny. AS074 was identified as *Chionaspis pinifoliae*. AS077 was identified as *Diaspidiotus osborni*. AS087 was identified via 16s phylogeny as *Diaspidiotus osborni* with a posterior probability of 52 while the 28s phylogeny identified it as *Dynaspidiotus californicus* with a posterior probability of 96. AS084 was identified as *Hemiberlesia rapax.* AS016, AS043, and AS027 had insignificant probability values to be identified as falling within a diaspidid phylogeny. Morphological analysis determined that these samples are likely in the family Halimococcidae. AS054 yielded an unknown 28s sequence. AS085 yielded an unknown 16s sequence. These samples will be further analyzed as to determine the exact species.

**Table 1:** Invasive status of original specimens based on sequence similarity to Genbank sequences. Invasive status was determined according to Miller *et al.*, 2005 [10]. An asterisk * indicates invasive status and a cross † indicates unknown invasive status. Dashed lines indicate no sequences were obtained for that particular specimen. For 16s sequences the host name was used instead of the symbiont name *Candidatus* Uzinura diaspidicola.

Sample #	28s	Accession Number	16s	Accession Number
	Phylogenetic ID		Phylogenetic ID	
AS013	Pseudalacaspis cockerelli*	KF887352	Pseudalacaspis cockerelli*	KF887383
AS015	Pseudalacaspis cockerelli*	KF887353	Pseudalacaspis cockerelli*	KF887384
AS016	undet Halimococcidae	KF887354	---	---
AS017	Pseudalacaspis cockerelli*	KF887355	Pseudalacaspis cockerelli*	KF887385
AS023	Pseudalacaspis cockerelli*	KF887356	Pseudalacaspis cockerelli*	KF887386
AS026	Pseudalacaspis cockerelli*	KF887357	Pseudalacaspis cockerelli*	KF887387
AS027	undet. Halimococcidae	KF887358	---	---
AS030	Pinaspis piperis*	KF887359	---	---
AS035	*Melanaspis obscura*	KF887360	*Melanaspis obscura*	KF887388
AS037	Carulaspis minima*	KF887361	Carulaspis juniperi*	KF887389
AS038	*Melanaspis obscura*	KF887362	*Melanaspis obscura*	KF887390
AS039	*Melanaspis obscura*	KF887363	---	---
AS040	*Melanaspis obscura*	KF887364	*Melanaspis obscura*	KF887391
AS041	*Melanaspis obscura*	KF887365	*Melanaspis obscura*	KF887392
AS042	Unaspis euonymi*	KF887366	---	---
AS043	undet Halimococcidae	KF887367	Unknown Symbiotic bacteria	KF887393
AS044	Pseudalacaspis cockerelli*	KF887368	Pseudalacaspis cockerelli*	KF887394
AS045	Pinaspis piperis*	KF887369	Pinaspis piperis*	KF887395
AS050	*Parlatoria sp.*	KF887370	---	---
AS053	Pseudalacaspis cockerelli*	KF887371	Pseudalacaspis cockerelli*	KF887396
AS054	†	KF887372	---	---
AS056	Pseudalacaspis cockerelli*	KF887373	Pseudalacaspis cockerelli*	KF887397
AS057	Pseudalacaspis cockerelli*	KF887374	Pseudalacaspis cockerelli*	KF887398
AS058	Pseudalacaspis cockerelli*	KF887375	Pseudalacaspis cockerelli*	KF887399
AS070	Carulaspis minima*	KF887376	Carulaspis juniperi*	KF887400
AS073	---	*---*	*Melanaspis obscura*	KF887401
AS074	*Chionaspis pinifoliae*	KF887377	*Chionaspis pinifoliae*	KF887402
AS077	---	*---*	*Diaspidiotus osborni†*	KF887403
AS079	---	*---*	*Melanaspis obscura*	KF887404
AS080	---	*---*	Fiorinia theae*	KF887405
AS081	Fiorinia theae*	KF887378	Fiorinia theae*	KF887406
AS082	---	*---*	Fiorinia theae*	KF887407
AS083	---	*---*	Fiorinia theae*	KF887408
AS084	Hemiberlesia rapax*	KF887379	---	---
AS085	---	*---*	†	KF887409
AS087	*Dynaspidiotus californicus*	KF887380	*Diaspidiotus osborni*†	KF887410
AS088	*Dynaspidiotus californicus*	KF887381	---	---
AS089	*Melanaspis obscura*	KF887382	*Melanaspis obscura*	KF887411
AS090	---	*---*	*Melanaspis obscura*	KF887412

Because cryptic diversity among armored scales still poses a challenge to growers, undertrained quarantine professionals and conservation biologists across the globe, alternative identification methods will improve diagnosis and control of such organisms. With the advent of cheaper sequencing and molecular methods, the technique we have presented here offers a potential method for accurate armored scale insect identification for individuals lacking a background in armored scale insect taxonomy. We offer the workflow below, as a guide for molecular identification of armored scale insects:

(1) Obtain DNA sequence of 28S and 16S from either in a lab utilizing the methods outlined above or by sending it to a contract lab.
(2) When accurate DNA sequences for one or both of the loci outlined above have been obtained, sequences should be imported into an already prepared, unaligned data file (FASTA file) in the form of a text document. The file is available for download through corresponding author’s faculty webpage and the unknown diaspidid sequences in question can be added after the end of the last sequence listed in the document. The sequence should be entered in this format: <28Suknown1, *i.e.*, left arrow, followed by the sequence name without spaces followed by a single return and the DNA sequence in all lowercase letters. Data files for both 16S and 28S genes can be copied from the following site, [20], under the publications tab, directly below citation for this publication.
(3) The entire data file should then be pasted in the data window on the online sequence alignment server, MAFFT [21]. In the segments below the window, the following settings should be selected. (a) **UPPERCASE**/**lowercase**—Amino Acid….. (b) **Direction of nucleotide sequences**—Adjust direction according to the first sequence (accurate for most cases) (c) **Output order**—aligned. An email address should be provided to MAAFT as well.
(4) Species identification can now by estimated three ways. (a) The output in MAFFT has two files to view. One is the aligned data set, which will have the most similar sequences next to each other. Viewing and comparing original data to provided data in the aligned data set can elucidate similarities between sequences. A high enough similarity infers a potential species match. (b) MAFFT also allows for viewing a rough phylogenetic tree. This tree depicts the sequences most closely related to the original data, and therefore provides an estimate of species identity. (c) The aligned data set can be exported from MAFFT and subjected to a rigorous phylogenetic analysis by uploading the data set into an online phylogenetics utility site such as CypresPortal and obtain a tree with higher specificity.

Once the identity of an armored scale is known, biologists can take appropriate measures to control it. Often such measures require species identification because the treatments are species specific, such as biological control efforts utilizing parasitoid wasps or pheromone traps [22,23]. A workable, easy protocol to identify common but taxonomically problematic scale insects will prove to be an important tool for many biologists lacking taxonomic expertise.

Beyond control of invasive or pest species, if those carrying out this protocol will share their data by uploading it to online databases such as Genbank [19], their collaborative efforts will help to elucidate the cryptic diversity of armored scale insects. As molecular systematists continue to work in this area, it is becoming increasing apparent that many of the cosmopolitan, invasive species of scale insects are actually many cryptic species making up a species complex [1,13,14]. Such complexes are of evolutionary significance and the data can be very useful across disciplines [24]. In this regard, scientists investigating evolutionary founded questions as well as those in applied fields such as agriculture can share data and collaborate in ways to move both fields in positive directions.

**Table 2:** Genbank sequences used in phylogenetic analysis. Accession numbers are provided. Dashed lines indicate a species was used in only one of the phylogenies. Double asterisks ** indicate the outgroup.

Sequence Name	Accession #	16s
	28s	
*Aonidiella aurantii*	---	GQ424836.1
*Aspidiotus nerii*	DQ145297.2	AY279402.1
*Aulacaspis difficilis*	DQ145298.2	DQ868801.1
*Carulaspis juniperi*	DQ145303.2	DQ868804.1
*Carulaspis minima*	DQ145302.2	GQ424837.1
*Chionaspis heterophylae*	GU349426.1	GQ424897.1
*Chionaspis pinifoliae*	GQ325459.1	DQ868807.1
*Chionaspis wistariae*	DQ145308.2	DQ868808.1
*Chrysomphalus dictyospermi*	DQ145310.2	GQ424913.1
*Diaspidiotus osborni*	---	GQ424913.1
*Diaspis coccois*	DQ145317.2	---
*Dynaspidiotus californicus*	DQ145322.2	---
*Fiorinia euryae*	---	DQ868822.1
*Fiorinia theae*	DQ145332.2	DQ868859.1
*Greeniella capitata*	---	GQ424922.1
*Hemiberlesia rapax*	DQ145344.2	GQ4249859.1
*Lindingaspis rossi*	GQ325507.1	GQ424933.1
*Megacanthaspis leucaspis*	DQ145359.2	GQ424869.1
*Melanaspis bromiliae*	DQ145360.2	DQ868835.1
*Melanaspis obscura*	GQ325511.1	GQ424850.1
*Mycetaspis personata*	DQ145366.2	DQ868838.1
*Nuculaspis californica*	GQ325515.1	GQ424839.1
*Parlatoria camelliae*	DQ145371.2	DQ868843.1
*Parlatoria theae*	DQ145373.2	DQ868841.1
*Pinnaspis piperis*	DQ145377.2	DQ868849.1
*Pinaspis strachani*	DQ145378.2	---
*Poliaspis syringae*	GQ325539.1	GQ424948.1
*Pseudalacaspis cockerelli*	DQ145384.2	DQ868851.1
*Pseudalacaspis pentagona*	DQ145385.2	DQ868852.1
Pseudococcus longispinus*	AY427400.1	JF714175.1
*Thysancoccus pandani*	DQ145391.2	GQ424871.1
*Unaspis euonymi*	DQ14593.1	DQ868855.1

## 4. Conclusions and Future Directions

Currently there are two large-scale molecular systematic studies of the family Diaspididae that are useful in determining species level identification of armored scale insects [13,15]. Both studies incorporate 28s data and Andersen *et al.* [13], also uses 16s data. Beyond these Gruwell *et al.* [17] were also able to demonstrate the congruence of a family level 16s + 23 rRNA bacterial genealogy to an armored scale insect genealogy using 28S rRNA and elongation factor 1-alpha. The genes used in this paper have been tested and found reliable for resolving species level phylogenies of armored scale insects. They are also known to be relatively simple to PCR and sequence reliably, thus making them efficient tools for molecular identification of armored scale insects for non-experts. We are aware of the DNA Barcode of Life effort utilizing a section of cytochrome oxidase one (CO1) as an identifier for all eukaryotic organisms and some armored scale insects are currently available in the BOLD (Barcode of Life Database) [25]. However, it is our experience that PCR and sequencing of this region of CO1 for armored scales is significantly more difficult and less reliable, thus in order to provide a workflow for non-molecular biologists to ID armored scale insects, we have chosen to pursue 28s and 16s rRNA.

Identification of armored scale insects via molecular biology technology looks hopeful, however, there are obstacles to overcome. Diversity is prevalent within most major genera of scales. With more sequences, identification of cryptic species within these genera should be elucidated. With the advent of new sequencing technologies and the continued expansion of online bioinformatics tools and databases, we hope to see an increase in contribution to scale insect DNA sequencing and identification that can lead to better diagnostics and control of invasive armored scale insects.
